# Efficient multiplex mutagenesis by RNA-guided *Cas9* and its use in the characterization of regulatory elements in the *AGAMOUS* gene

**DOI:** 10.1186/s13007-016-0125-7

**Published:** 2016-04-25

**Authors:** Wenhao Yan, Dijun Chen, Kerstin Kaufmann

**Affiliations:** Institute for Biochemistry and Biology, Potsdam University, 14476 Potsdam, Germany

**Keywords:** RNA-guided *Cas9*, Multiplex mutagenesis, Large fragment deletion, Germline transmission

## Abstract

**Background:**

The efficiency of multiplex editing in plants by the RNA-guided *Cas9* system is limited by efficient introduction of its components into the genome and by their activity. The possibility of introducing large fragment deletions by RNA-guided *Cas9* tool provides the potential to study the function of any DNA region of interest in its ‘endogenous’ environment.

**Results:**

Here, an RNA-guided *Cas9* system was optimized to enable efficient multiplex editing in *Arabidopsis thaliana*. We demonstrate the flexibility of our system for knockout of multiple genes, and to generate heritable large-fragment deletions in the genome. As a proof of concept, the function of part of the second intron of the flower development gene *AGAMOUS* in *Arabidopsis* was studied by generating a Cas9-free mutant plant line in which part of this intron was removed from the genome. Further analysis revealed that deletion of this intron fragment results 40 % decrease of *AGAMOUS* gene expression without changing the splicing of the gene which indicates that this regulatory region functions as an activator of *AGAMOUS* gene expression.

**Conclusions:**

Our modified RNA-guided *Cas9* system offers a versatile tool for the functional dissection of coding and non-coding DNA sequences in plants.

**Electronic supplementary material:**

The online version of this article (doi:10.1186/s13007-016-0125-7) contains supplementary material, which is available to authorized users.

## Background

In recent years, the gene editing technology has been intensively developed, featured by the application of sequence specific nucleases, including zinc finger nucleases (ZFN), transcription activator-like effector nucleases (TALENs) and the RNA-guided CRISPR-ASSOCIATED 9 (*Cas9*) nuclease. The latter was derived from the Clustered Regularly Interspaced Short Palindromic Repeats (CRISPR) system which acts as in adaptive immune response in bacteria and archaea [1–4].

CRISPR/Cas9 system was discovered as bacterial type II defense system which consists of *Cas9* nuclease and two non-coding RNAs; trans-activating crRNA (tracrRNA) and a precursor crRNA (pre-crRNA). Pre-crRNA contains an array of short sequences that are derived from pathogens and later guide the crRNA/tracrRNA/CAS9 complex to the target sequence. The target sequence needs to be complementary to the guide sequence and an adjacent NGG motif which is called protospacer adjacent motif (PAM) is required to be present [2–4]. Adopting this defense machinery from bacteria, the RNA-guided *Cas9* gene editing tool was developed [5–7]. The system combines the guide sequence and the chimeric tracrRNA/crRNA unit into a single guide RNA (sgRNA), and additionally contains a *Cas9* expression cassette. This tool has been reported to efficiently enable genome editing in human cells, in various other animal species and also in plants [8–12].

Although the two major components, sgRNA and *Cas9* can be directly injected into plant cells [13, 14], conventional T-DNA based transformation is still the most common and simple way to deliver sgRNA and *Cas9* into the plant. This limits the capacity of the system in plants due to low sgRNA/*Cas9* delivering efficiency [15]. Several studies aiming to establish an efficient RNA-guided *Cas9* tool or to probe the inheritance of *Cas9* caused mutation in plants were carried out, especially with the scope of generating stable transgenic lines [16]. Feng et al. generated mutations in rice and in *Arabidopsis* by engineering a CaMV 35S driven human codon optimized *Cas9* and sgRNA expression cassette into a binary vector [17]. By driving the expression of Cas9 under the promoter of *INCURVATA2* (*ICU2*) gene to disturb the function of *FT*, nine out of eleven T1 transformants already showed *ft* phenotype which is late flowering [18]. Ma et al. managed to simultaneously target eight genes in rice but did not get high efficiency when targeting genes in *Arabidopsis* [19]. By expressing sgRNAs in a designed polycistronic tRNA/gRNA (*PTG*) gene, Xie et al. simultaneously targeted eight loci in rice protoplasts [20]. Another study reported the use of the promoter of an egg-cell specific gene, *EC1.2* to drive *Cas9* expression in order to mutagenize three genes. Two out of twenty-four transformants showed a triple-mutant phenotype in the first generation [21].

A particularly interesting application of CRISPR/Cas9 targeted mutagenesis is the generation of large-fragment deletions, because this enables easy PCR-based genotyping of the mutations. So far, the efficiency of germline transmission of large-fragment deletions has not been analyzed in plants. In this study, we optimized a RNA-guided *Cas9* system by driving the expression of *Arabidopsis* codon optimized *Cas9* (pco*Cas9*) gene under the promoter of *UBIQUTIN 10* (*UBQ10*) which highly expressed in early embryos, and was also active later in plant development. By simply rearranging the order of restriction enzymes, multiple sgRNAs could be easily combined within one single vector thus to allow multiplex targeting. Compared with the system in which *Cas9* gene was under the control of CaMV35S promoter, the efficiency of multiplex targeting was strikingly increased. We found that the key to increase the efficiency of the RNA-guided *Cas9* system in *Arabidopsis* is to express *Cas9* in meristems and embryonic cells at high levels. A 5.19 kb deletion of a specific genomic region was produced efficiently using our optimized system and this large deletion was inherited to the next generation via germline transmission. Using RNA-guided *Cas9* method, the function of a 450 bp regulatory sequence in the second intron of the *AGAMOUS* gene was studied by deleting this fragment from the genome. We found that this regulatory region acted as an activator of *AGAMOUS* gene expression predominantly in early arising flowers, without affecting the splicing of the transcript.

## Results

### Improving the efficiency of multiplex editing by optimized activity of *CAS9* protein

**Fig. 1:**
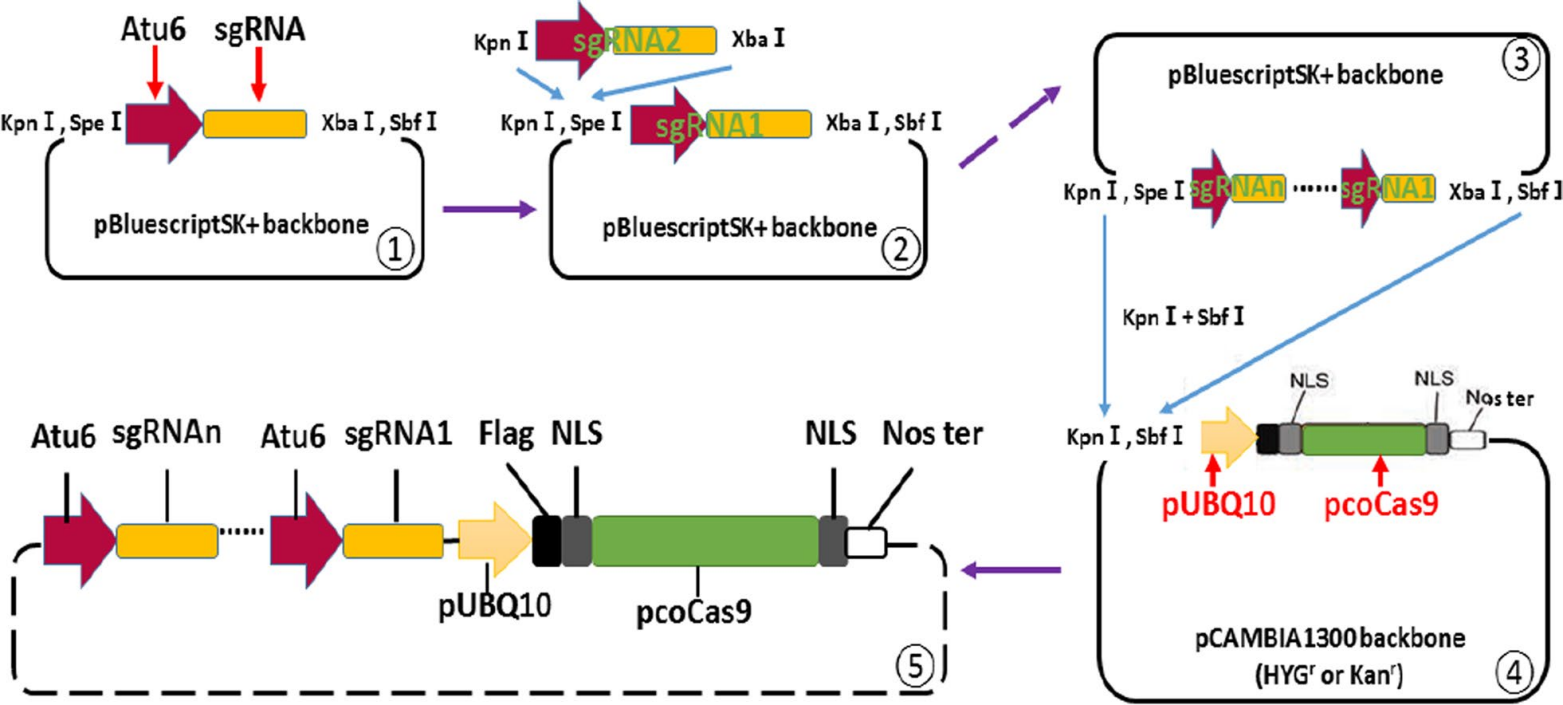
A newly developed RNA-guided *Cas9* system. Diagrams depict modification of a previously reported RNA-guided *Cas9* tool to an efficient multiplex editing vector system. Two adjacent cutting sites, *KpnI* and *SpeI,* and two other closely linked cutting sites, *XbaI* and *SbfI,* are located at 5′ and 3′ end of the sgRNA expression cassette, respectively (①). Two independent sgRNA expression cassettes can be combined when one of them is digested by *KpnI* and *SpeI* while the other one is digested by *KpnI* and *XbaI* (②). More sgRNA expression cassettes can be combined in one plasmid by repeating the same procedure (③). The combined sgRNA expression cassettes containing different guide sequences against different loci can be entirely isolated by cutting with *KpnI* and *SbfI* by which the binary vector containing the Cas9 expression cassette was also digested (④, ⑤). The *UBIQUTIN 10* (*UBQ10*) promoter was used to drive the expression of *Arabidopsis* codon optimized Cas9 (pcoCas9) gene (④). pUBQ10: promoter of *Arabidopsis UBIQUTIN* 10 (*UBQ10*; *AT4G05320*) gene; pcoCas9: *Arabidopsis* codon optimized *Cas9* gene; Atu6: any of Arabidopsis U6-1, U6-26 or U6-29 gene promoters

We initially utilized a previously established RNA-guided *Cas9* system using the *Arabidopsi*s U6-26 (AtU6-26) promoter and a 2 × 35S promoter to drive expression of the sgRNA scaffold and human codon-optimized *Cas9* (*hucoCas9*) gene, respectively [17]. This plasmid series provided starting material to generate a RNA-guided *Cas9* tool for multiplex editing in plants. In order to express multiple gRNAs in one single vector, we modified the system by introducing *Spe*I and *Xba*I restriction sites that generate compatible cohesive ends, in order to express multiple gRNAs from one single vector (Fig. 1).

**Fig. 2:**
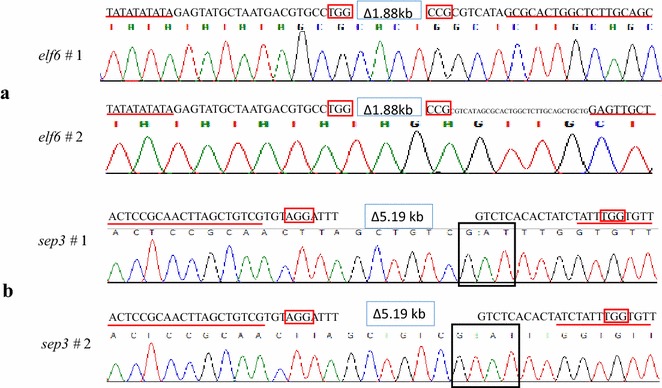
Large fragment mutation in *ELF6* and *SEP3* genes. Two *elf6* mutated alleles (**a**) and two *sep3* mutated alleles (**b**) were confirmed by sequencing. *Red boxes* show the PAM motifs of the target region of a certain guide sequence and the *red underlined* sequence indicates the sequences maintained after deleting a big fragment; number means the length of fragment that is too long to visualize

To test the possibility to produce large fragment deletions using the newly developed multiple gRNA expression system, two gRNA were expressed to target the flowering time regulator *EARLY FLOWERING 6* (*ELF6*) [22] gene to generate a 1.88 kb deletion and another two gRNAs were designed to remove the 5.1 kb entire *SEPALLATA3* (*SEP3*) locus [23]. For convenience, “mutation” hereafter refers to a deletion that is large enough to be detected as length polymorphism by a standard PCR approach. Among 50 independent T1 plants targeting *ELF6*, two individuals contain the mutated *elf6* allele (Fig. 2a). In the case of *SEP3*, two out of 31 T1 plants were shown to have the expected mutation, which were confirmed by sequencing (Fig. 2b).

We then tested the efficiency of multiplex mutagenesis using this system. Two sgRNAs which target the *RELATIVE OF EARLY FLOWERING 6* (*REF6*) gene and two more sgRNAs targeting the *At5g46910* gene were added into the previous *ELF6* gene targeting construct to generate a new construct, *ELF6/REF6/At5g46910*_p35ShucoCas9. *REF6* is the closest homolog of *ELF6* in the Arabidopsis genome [22]. The *At5g46910* gene is closely related with *REF6* and *ELF6*, and has been suggested to be potentially functionally redundant with both genes [24, 25]. We did not detect any mutation for any of the three genes from 48 *ELF6/REF6/At5g46910*_p35ShucoCas9 transformants (Table 1).

**Table 1:** Efficiency of multiplex editing by using different Cas9 expression cassettes

Target gene (s) (construct)	# Plants	*elf6*	*ref6*	*at5g46910*	*Triple*
		# Plants	# Plants	# Plants	# Plants
*ELF6*/*REF6*/*At5g46910* (p35S::hucoCas9)	48	0	0	0	0
*ELF6*/*REF6*/*At5g46910* (p35S::pcoCas9)	41	3	2	0	0
*ELF6*/*REF6*/*At5g46910* (pUBQ10::hucoCas9)	48	14	12	7	6
*ELF6*/*REF6*/*At5g46910* (pUBQ10::pcoCas9)	48	5	22	12	5

Leaf samples from T1 transformants were genotyped by PCR. Mutation was scored when a band with expected deletion was obtained. Triple-mutation (*triple*) means that with the same plant, mutated bands for all the three loci could be detected. #, number of

As each sgRNA expression cassette has its own U6 promoter which should ensure high gRNA expression level, we speculated that the lower efficiency was caused by competition of the gRNAs for CAS9 protein. In addition, since the mutation made by RNA-guided *Cas9* is somatic, high expression of *Cas9* in meristem and during embryo development might help to increase the mutagenesis efficiency. To test this hypothesis, we made several modifications to the system. First, the human codon optimized *Cas9* (*hucoCas9*) was replaced by *Arabidopsis* codon optimized *Cas9* (*pcoCas9*) which was reported to produce two times more CAS9 protein in *Arabidopsis* cells than the human optimized one when the same promoter system was used [8]. Also, a *UBIQUTIN 10* (*UBQ10*) promoter was used to drive the expression of either *hucoCas9* or *pcoCas9* gene (Fig. 1). *UBQ10* is a constitutively active gene, and it is especially highly expressed in early embryos (Additional file 1: Fig. S1), in which CaMV35S promoter does not work efficiently [26, 27]. All the three new constructs contain the same three pairs of gRNAs, targeting *ELF6*, *REF6* and *At5g46910*, respectively. As shown in Table 1, with the original combination of 35S promoter and *hucocas9* (p35S::*hucoCas9*), no mutated allele (with large fragment deletion) was detected by PCR among 48 T1 transformants. When *hucoCas9* was replaced by *pcoCas9* under the 35S promoter, three plants had *ELF6* mutated alleles and two plants had mutation in the *REF6* gene but none showed mutation in *At5g46910* gene out of 41 T1 transformants. Next, we tested the impact of replacing the 35S promoter by a *UBQ10* promoter. Strikingly, 14 plants were identified as mutant for *ELF6*, 12 mutated *REF6* and seven plants with mutated *At5g46910* alleles could be detected from 48 transformants, when *hucoCas9* was driven by *UBQ10* promoter (pUBQ10::*hucoCas9*). The number of *REF6* and *At5g46910* mutated plants increased further to 22 and 12, respectively when *pcoCas9* was driven by the *UBQ10* promoter (pUBQ10::*pcoCas9*). The results show that our optimized RNA-guided *Cas9* system has improved efficiency for multiplex mutagenesis. More importantly, our data strongly support the notion that in *Arabidopsis*, both the CAS9 protein dosage and the expression of *Cas9* in meristematic and early embryonic cells are important for mutagenesis efficiency of the RNA-guided *Cas9* system.

### Germline transmission of large fragment deletions

**Fig. 3:**
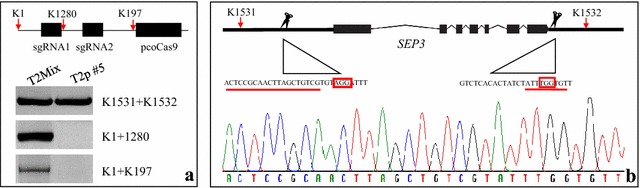
Recovery of a 5.19 kb deletion in a *Cas9*-free T2 plant. Detection of mutated allele and the absence of *Cas9* T-DNA by PCR (**a**). The PCR products produced by K1531 and 1532 were analyzed by sequencing (**b**). The chromatogram of the sequencing result confirms the mutation. T2p#5: plant #5 of a T2 population

As long as *Cas9* is expressed, new mutations can be produced. It is therefore impossible to distinguish these newly generated (somatic) mutations from a mutation transmitted through germ cells. Therefore, one major concern with applying RNA-guided *Cas9* tool in plants is the transmission of mutations to the next generation, especially in the cases of large fragment deletions which have lower mutagenesis efficiency. To test the germ line transmission efficiency of large fragment mutations produced by the RNA-guided *Cas9* introduced here, we firstly generated a *SEP3* full gene deletion (5.19 kb) by using *UBQ10* driven *pcoCas9*. We observed a much higher mutation rate (8 out of 29 T1 transformants) than with the 35S promoter (2 out of 31 T1 plants), which was in line with the results above showing that expression of *pcoCas9* from the *UBQ10* promoter can largely increase the mutagenesis efficiency. To ensure that the mutation in T2 plants was due to germline transmission, only T2 plants in which *Cas9* T-DNA was not present any longer were selected for analysis. We obtained 64 *Cas9*-free plants from 300 individuals of a T2 population. One plant (plant #5) was found to possess the expected mutation and to be T-DNA free (Fig. 3a, b). This result showed that a large fragment deletion was efficiently produced by our RNA-guided *Cas9* system and successfully transmitted to the next generation.

### Dissecting the biological role of a 450 bp regulatory region in the *AGAMOUS* second intron

**Fig. 4:**
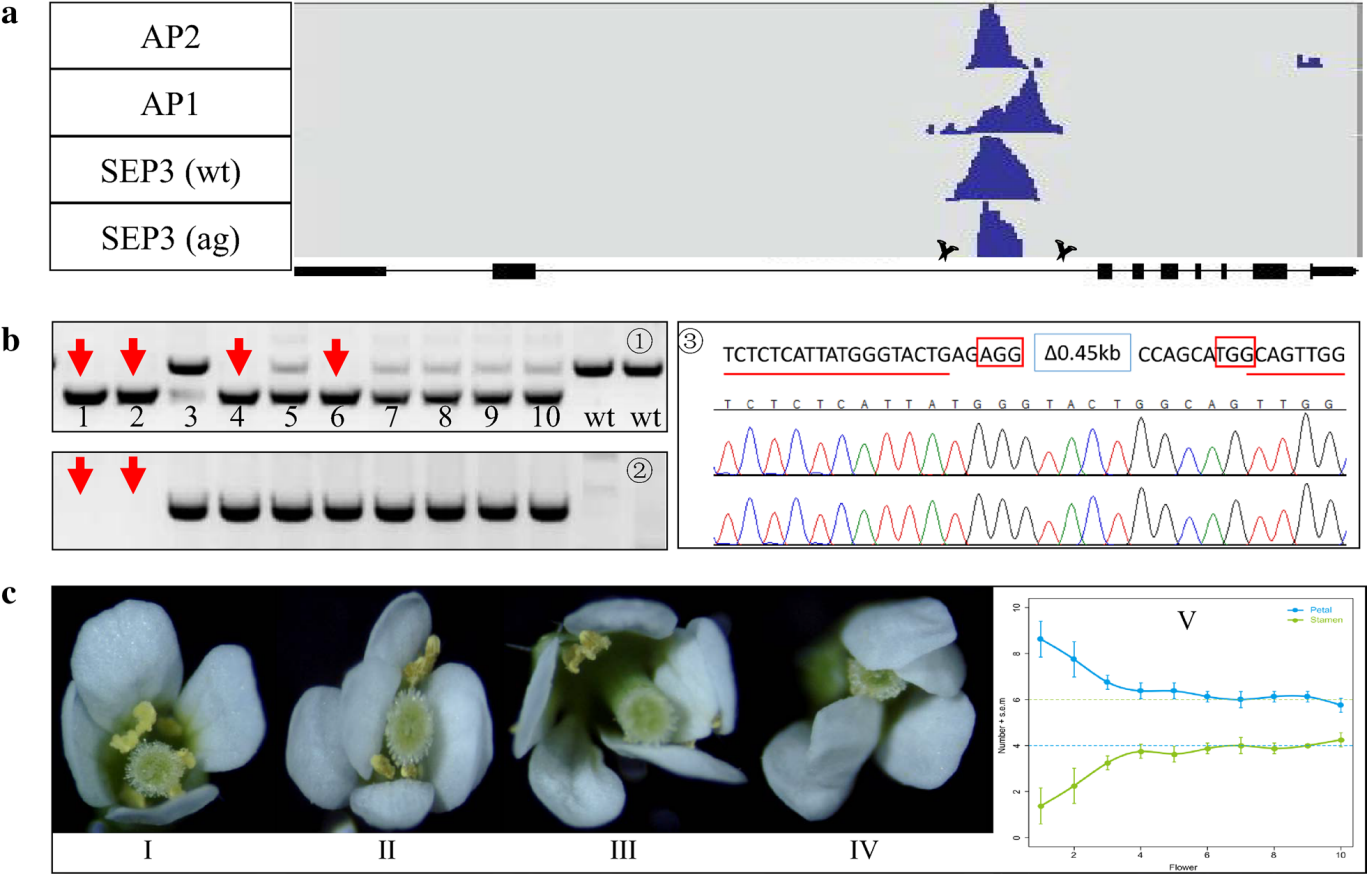
Functional analysis of a regulatory region in the second intron of *AGAMOUS*. **a** Binding profiles of MADS-domain transcription factors (TFs) in the second intron of *AGAMOUS*. *Cross symbols* indicate the expected cutting regions. **b** Ten individuals from the progeny of a T1 plant were genotyped for presence of mutation (①) and T-DNA (②). The mutation was confirmed by sequencing (③). *Red boxes* in ③ indicate PAM motifs. **c** Homozygous mutant shows partial homeotic transformations of stamens to petals (II–V, I is wild type flower)

**Fig. 5:**
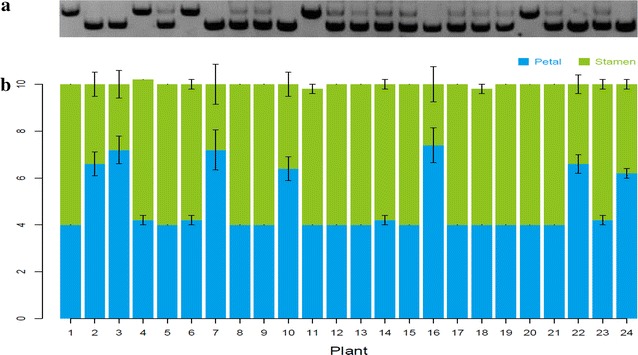
The 450 bp deletion in the *AG* 2nd intron is co-segregating with the flower mutant phenotype. **a** The genotypes of 24 individuals of an F2 population. **b** Quantification of petal and stamen numbers. The number of petal and stamens from first five flowers of a plant were counted. Data were shown as mean ± SE

**Fig. 6:**
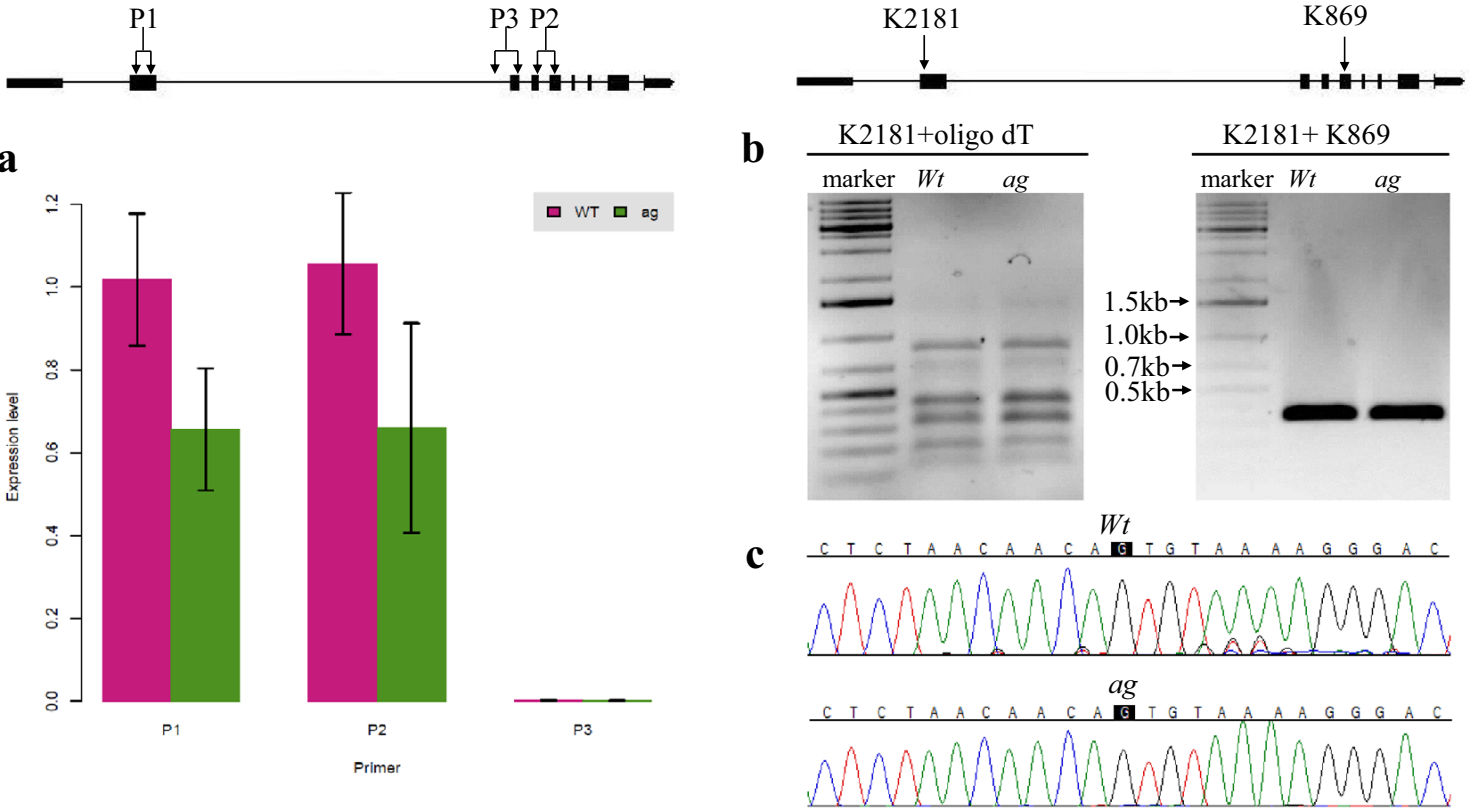
The 450 bp deletion in the *AG* 2nd intron results in reduction of *AG* expression without interfering the splicing of the gene. **a** *AG* expression in wild type and mutant was detected by three pairs of primers shown in the *upper scheme*. The expression data represents three biological replicates and three technical replicates. Data were shown as mean ± SD. **b** Agarose gel pictures of products of two rounds of PCR, a reverse transcription PCR using oligodT primer as one of the primers followed by a nested PCR using a more internal reverse primer. **c** Sequencing results of the products from the second round of PCR in wild type and mutant

The possibility to efficiently delete a specific sequence from the genome makes it possible to study the function of non-coding DNA sequences, such as long non-coding RNA loci, enhancers, introns and UTRs, in their native context. *AGAMOUS* (*AG*) is a floral homeotic C class gene which is responsible for stamen and carpel specification in *Arabidopsis* [28]. The *AG* second intron was reported to contain several regulatory regions which are important for *AG* gene expression [29, 30]. Especially, the 3′ region of the intron was found to be bound in vivo by flower developmental regulators including AP1, AP2 and SEP3. The transcription factor binding sites are located within a region of 450 bp in length (Fig. 4a) [31]. The function of the region has previously been analyzed using transgenic reporter gene analyses [30], but never in the ‘endogenous’ genomic context. In order to probe the function of this genomic region, we designed two gRNAs which aimed to remove this sequence from the locus. Of 12 T1 transformants, three plants (plants #3, #6 and #10) showed a PCR fragment size indicating the deletion (Additional file 1: Fig. S2). Plants #3 and #10 were chosen for testing of the progeny. A mild *ag*-like mutant phenotype, characterized by flowers with partial homeotic transformations of stamens to petals, could be observed from the progeny populations of both T1 lines. We focused on the progeny population of plant #3. Four individuals of ten T2 lines showed a homozygous mutant genotype and two of the four plants no longer have the *Cas9* T-DNA (Fig. 4b, panels ① and ②). Further sequencing results on the* Cas9* T-DNA free plants confirmed the deletion of the target genomic region (Fig. 4b, panel ③). All of the 30 individuals from a T3 population derived from *Cas9*-free T2 plant #1 showed partial homeotic transformations of stamens to petals. However, carpel development was not affected by this mutation (Fig. 4c, panels II, III and IV). Interestingly, the first flowers of an inflorescence showed more dramatic flower abnormalities then the later ones (Fig. 4c, panel V). In order to further confirm that the phenotype was caused by the deletion of the 450 bp intron fragment, the *Cas9*-free plant #1 was back-crossed with Col-0 to generate an F2 population. In an F2 population which consisted of 24 individuals, only seven homozygous mutants showed homeotic transformations of stamens to petals (Fig. 5). In order to investigate how the 450 bp deletion affects *AGAMOUS* expression and whether the deletion affects the splicing of *AGAMOUS* mRNA, three pairs of primers locating at the beginning of the transcribed region, exactly at the splicing site and in an exon downstream of the splicing site were used to detect the *AG* expression. The results of reverse transcription (RT)-qPCR show that *AG* expression is reduced to 60 % in the mutant compared to wild type, and no alternatively spliced transcripts can be detected in wild type and mutant (Fig. 6a). This was further confirmed by RT-PCR results in wild type and mutant, using the primer which binds to the beginning of the gene transcript together with an oligo dT primer, followed by re-amplification using a reverse primer against the downstream exon of the splicing site. Only a PCR product of one specific size could be observed from each genotype (Fig. 6b). Sequencing revealed that this PCR product represents the same gene transcript in wild type and mutant (Fig. 6c). The results above indicate that the function of this 450 bp regulatory region is important for the activation of *AG* without affecting the splicing of the gene, particularly in early arising flowers in the inflorescence. Our study here provides a nice example that the RNA-guided *Cas9* system can accelerate the functional dissection of non-coding DNA regions in the plant genome in an endogenous context.

## Discussion

Efforts have been made to increase the efficiency of the RNA-guided *Cas9* system in plants by modulating the *Cas9* activity. Major attempts including the one we described here focus on using different promoters to enable CAS9 working in the most optimal cells and tissues, such as the *ICU2* or the *EC1.2* promoters [18, 21]. A similar study showed that the rate of heritable mutations can be significantly increased by expressing Cas9 under germ-line cell specific promoters [32]. In the study presented here, we expressed *Cas9* at high level in meristematic cells and at early stages of embryo development by using the *UBQ10* promoter. Compared with 35S driven *Cas9* expression, the efficiency of large fragment deletion increased by at least three times (in case of mutating the *ELF6* gene). During the preparation of this manuscript, a promoter that is specifically active in meristematic tissues, the *YAO* promoter, was reported to improve editing efficiency in *Arabidopsis* [33]. In this study, a similar approach to combine multiple gRNAs by taking the advantage of isocaudomer enzymes and to drive the expression of *Cas9* in cells with strong differentiation activity was applied. These, as well as our studies achieved higher mutagenesis efficiency higher than the original system based on the 35S promoter. In addition, the efficiency was further enhanced by using *Arabidopsis* codon optimized *Cas9*. The impact of choosing a promoter that is especially highly expressed in meristematic cells should also receive attention when attempting to increase the efficiency of the RNA-guided *Cas9* system in other plant species. In crops such as rice and maize, transformation with embryonic callus is the most efficient way to deliver T-DNA into cells. Promoters that are highly expressed in callus might help to increase the efficiency of RNA-guided *Cas9* system in terms of generating heritable mutations. Due to the conserved function of *ICU2* promoter and ubiquitin genes, promoters of *ICU2* and *UBQ10* homologs could be candidates.

By expressing two gRNAs targeting a 450 bp region in the second intron of *AG* gene, the fragment was successfully removed from the genome and *Cas9* T-DNA-free transgenic lines were obtained. This regulatory region was previously shown to be important for the activation of *AG* expression in stage 3 floral meristems using transgenic GUS reporter assays [30]. Indeed, the enhancer mutant plants showed a phenotype that can be linked with lower *AG* activity, which indicates that this intron fragment is important for activating *AG* expression. However, compared with the classical *ag* loss of function mutant [28, 34], the mutant we generated shows a much weaker phenotype and has almost normal seed productivity. We confirmed that deletion of the 450 bp intron fragment resulted in around 40 % reduction of *AG* gene expression without affecting the splicing of the gene. The phenotype was most pronounced in early arising flowers, which is not expected based on the classical transgenic analyses [30] and can be explained by two alternative mechanisms. Either this enhancer region is especially required for efficient activation of *AG* in flowers that arise soon after floral transition, while in later flowers, other redundantly acting enhancers may compensate or the repressor levels are decreased. Alternatively, higher levels of *AG* activity may be required in early arising flowers than in later arising ones to specify the reproductive organs, in particular stamens. In any case, the analysis of this regulatory region in its native context provides novel functional insights that offer a starting point for future functional analyses. Our results therefore show that besides studying gene functions, RNA-guided *Cas9* has the unique potential to elucidate the roles of non-coding DNA regions in their native context. The only potential limitation in regulatory element analysis is that CAS9 needs the presence of PAM motif in the cutting position. Recently, it was found that PAM sequence specificity can be altered which provides more flexibility in target choice [35].

To exclude the effect of somatic mutation by *Cas9* in next generation, we selected *Cas9*-free plants from the progeny of a confirmed T1 transformant for analysis. The number of plants needed for selecting a *Cas9*-free plants is largely determined by the transmission efficiency of a mutation to next generation. A 5.19 kb mutation was successfully transmitted to next generation (1 individual out of 64 plants) while for the mutation of deleting 450 bp fragment from *AG* second intron, only from ten plants, two mutants were *Cas9*-free. Selection of *Cas9*-free plants produces a mutant without T-DNA-insertion, which could be a big advantage for future molecular breeding.

## Conclusions

Through modifying a previously published RNA-guided *Cas9* system, high mutagenesis efficiency was achieved for either mutating multiple genes simultaneously or for generating large fragment deletions. According to the observation that mutagenesis efficiency was greatly enhanced by driving *Cas9* expression in embryonic/meristematic cells, we propose that the key to optimize the RNA-guided *Cas9* system for higher efficiency is to select a promoter that ensures high expression of *Cas9* in embryonic cells. The function of part of the second intron of the flower developmental gene *AG* in *Arabidopsis* was verified to be required for *AG* activation by successfully generating a *Cas9*-free mutant plant line in which part of this intron was removed from the genome. This result demonstrates the ability of RNA-guided *Cas9* to facilitate functional analysis of coding and non-coding DNA sequences in plants.

## Methods

### Plasmids and plant material

The original sgRNA expressing plasmid obtained from Feng et al. [17] was modified to have *KpnI*/*SpeI* tandem cutting sites at the beginning of Atu6-26 promoter and *XbaI*/*SbfI* sites after polyT terminator. This plasmid allows the combination of multiple gRNAs. In order to simplify the procedure to make RNA-guided *Cas9* constructs, *Cas9* was integrated to the binary vector pCAMBIA1300.All the sgRNA primers were designed with the web-tool “CRISPR PLANT” (http://www.genome.arizona.edu/crispr/) [36]. Detailed methods for modifying the RNA-guided *Cas9* system and for making constructs mentioned in this study is described in Additional file 1. The transgenic lines were produced by infiltrating Col-0 using floral-dip method [37].

### Genotyping and phenotyping

Seeds from infiltrated plants were selected by antibiotics (hygromycin or kanamycin). Surviving plants were transplanted into soil. After recovering for two to three weeks, three or four leaves from the same plants were pooled as one sample for genotyping. Two primers, one of which locates around 500 bp away from each of the target site, respectively, were designed for PCR-based genotyping. The PCR products were purified from agarose gel and sent directly for sequencing, if there was an expected band for mutated allele. The absence of sgRNA-*Cas9* in a plant was confirmed by PCR using the primers against T-DNA insertion. The progeny of T-DNA-free candidates were also subject to antibiotic selected except for the *SEP3* case. All the PCR templates and PCR reactions were prepared by using Phire plant direct PCR master mix kit (Thermo Scientific, USA) following the manufacturer’s instructions. The photos of entire plants or plates were taken by camera (NEX-5R, SONY, Japan), and a stereo-microscope (Discovery, Zeiss, Germany) was used for photographing single flowers.

### Expression analysis

The inflorescence of four weeks’ old Col-0 and *ag* mutant in which the 450 bp intron region was deleted were harvested for RNA extraction. Total RNA was extracted by the Trizol method (Sigma, USA) according to the manufacturer’s instructions and then 1 μg total RNA was subjected to cDNA synthesis using ProtoScript first Strand cDNA synthesis kit (NEB, United Kingdom) after DnaseI treatment (NEB, United Kingdom). Real-time PCR was performed with the SsoAdvanced Universal SYBR green Supermix on a CFX CONNECT real-time PCR system (BioRad, USA). Expression data were collected in three biological replicates with three technological replicates for each biological repeat. The Tip41-like gene (AT4G34270) was used as reference gene for normalization. Sequence information for all the primers used here could be found in Additional file 1: Table S1.

## Additional file

10.1186/s13007-016-0125-7 Expression pattern of the *AT4G05320* (*UBQ10*) gene in *Arabidopsis* (**Fig. S1**). **a** shows spatial and temporal expression pattern of *AT4G05320* gene during *Arabidopsis* seed development and stages of the life cycle and **b** indicates distribution of expression level of *AT4G05320* gene. *Heat maps* (*left*) show the normalized microarray expression value according to the color scales shown. *Bar chart* (*right*) shows the distribution of expression levels in different tissues and in different development stages. Genotyping of 12 T1 transformants for deleting a 450 bp intron segment in the *AGMAMOUS* gene (**Fig. S2**). The size of mutated allele is 450 bp smaller than the wild type allele. Plants #3, #6 and #10 in the *black box* contain the mutated allele. Primers used in this study and procedure for modifying the CRISPR/Cas9 system and protocol to construct a CRISPR/Cas9 vector.

## References

[1] Gaj T, Gersbach CA, Barbas CF (2013). ZFN, TALEN, and CRISPR/Cas-based methods for genome engineering. Trends Biotechnol.

[2] Wiedenheft B, Sternberg SH, Doudna JA (2012). RNA-guided genetic silencing systems in bacteria and archaea. Nature.

[3] Horvath P, Barrangou R (2010). CRISPR/Cas, the immune system of bacteria and archaea. Science.

[4] Sorek R, Lawrence CM, Wiedenheft B (2013). CRISPR-mediated adaptive immune systems in bacteria and archaea. Annu Rev Biochem.

[5] Jinek M, Chylinski K, Fonfara I, Hauer M, Doudna JA, Charpentier E (2012). A programmable dual-RNA-guided DNA endonuclease in adaptive bacterial immunity. Science.

[6] Cong L, Ran FA, Cox D, Lin S, Barretto R, Habib N, Hsu PD, Wu X, Jiang W, Marraffini LA (2013). Multiplex genome engineering using CRISPR/Cas systems. Science.

[7] Mali P, Yang L, Esvelt KM, Aach J, Guell M, DiCarlo JE, Norville JE, Church GM (2013). RNA-guided human genome engineering via Cas9. Science.

[8] Li JF, Norville JE, Aach J, McCormack M, Zhang D, Bush J, Church GM, Sheen J (2013). Multiplex and homologous recombination-mediated genome editing in *Arabidopsis* and *Nicotiana benthamiana* using guide RNA and Cas9. Nat Biotechnol.

[9] Nekrasov V, Staskawicz B, Weigel D, Jones JD, Kamoun S (2013). Targeted mutagenesis in the model plant *Nicotiana benthamiana* using Cas9 RNA-guided endonuclease. Nat Biotechnol.

[10] Li W, Teng F, Li T, Zhou Q (2013). Simultaneous generation and germline transmission of multiple gene mutations in rat using CRISPR-Cas systems. Nat Biotechnol.

[11] Bortesi L, Fischer R (2015). The CRISPR/Cas9 system for plant genome editing and beyond. Biotechnol Adv.

[12] Hsu PD, Lander ES, Zhang F (2014). Development and applications of CRISPR-Cas9 for genome engineering. Cell.

[13] Svitashev S, Young JK, Schwartz C, Gao H, Falco SC, Cigan AM (2015). Targeted mutagenesis, precise gene editing, and site-specific gene insertion in maize using Cas9 and guide RNA. Plant Physiol.

[14] Woo JW, Kim J, Kwon SI, Corvalan C, Cho SW, Kim H, Kim SG, Kim ST, Choe S, Kim JS (2015). DNA-free genome editing in plants with preassembled CRISPR-Cas9 ribonucleoproteins. Nat Biotechnol.

[15] Belhaj K, Chaparro-Garcia A, Kamoun S, Patron NJ, Nekrasov V (2015). Editing plant genomes with CRISPR/Cas9. Curr Opin Biotechnol.

[16] Belhaj K, Chaparro-Garcia A, Kamoun S, Nekrasov V (2013). Plant genome editing made easy: targeted mutagenesis in model and crop plants using the CRISPR/Cas system. Plant Methods.

[17] Feng Z, Zhang B, Ding W, Liu X, Yang DL, Wei P, Cao F, Zhu S, Zhang F, Mao Y (2013). Efficient genome editing in plants using a CRISPR/Cas system. Cell Res.

[18] Hyun Y, Kim J, Cho SW, Choi Y, Kim JS, Coupland G (2015). Site-directed mutagenesis in *Arabidopsis thaliana* using dividing tissue-targeted RGEN of the CRISPR/Cas system to generate heritable null alleles. Planta.

[19] Ma X, Zhang Q, Zhu Q, Liu W, Chen Y, Qiu R, Wang B, Yang Z, Li H, Lin Y (2015). A robust CRISPR/Cas9 system for convenient, high-efficiency multiplex genome editing in monocot and dicot plants. Mol Plant.

[20] Xie K, Minkenberg B, Yang Y (2015). Boosting CRISPR/Cas9 multiplex editing capability with the endogenous tRNA-processing system. Proc Natl Acad Sci USA.

[21] Wang ZP, Xing HL, Dong L, Zhang HY, Han CY, Wang XC, Chen QJ (2015). Egg cell-specific promoter-controlled CRISPR/Cas9 efficiently generates homozygous mutants for multiple target genes in *Arabidopsis* in a single generation. Genome Biol.

[22] Noh B, Lee SH, Kim HJ, Yi G, Shin EA, Lee M, Jung KJ, Doyle MR, Amasino RM, Noh YS (2004). Divergent roles of a pair of homologous jumonji/zinc-finger-class transcription factor proteins in the regulation of *Arabidopsis* flowering time. Plant Cell.

[23] Pelaz S, Ditta GS, Baumann E, Wisman E, Yanofsky MF (2000). B and C floral organ identity functions require *SEPALLATA* MADS-box genes. Nature.

[24] Lu F, Cui X, Zhang S, Jenuwein T, Cao X (2011). *Arabidopsis REF6* is a histone H3 lysine 27 demethylase. Nat Genet.

[25] Crevillen P, Yang H, Cui X, Greeff C, Trick M, Qiu Q, Cao X, Dean C (2014). Epigenetic reprogramming that prevents transgenerational inheritance of the vernalized state. Nature.

[26] Ouwerkerk PB, de Kam RJ, Hoge JH, Meijer AH (2001). Glucocorticoid-inducible gene expression in rice. Planta.

[27] Sunilkumar G, Mohr L, Lopata-Finch E, Emani C, Rathore KS (2002). Developmental and tissue-specific expression of CaMV 35S promoter in cotton as revealed by GFP. Plant Mol Biol.

[28] Bowman JL, Smyth DR, Meyerowitz EM (1989). Genes directing flower development in *Arabidopsis*. Plant Cell.

[29] Sieburth LE, Meyerowitz EM (1997). Molecular dissection of the *AGAMOUS* control region shows that cis elements for spatial regulation are located intragenically. Plant Cell.

[30] Deyholos MK, Sieburth LE (2000). Separable whorl-specific expression and negative regulation by enhancer elements within the *AGAMOUS* second intron. Plant Cell.

[31] Kaufmann K, Wellmer F, Muino JM, Ferrier T, Wuest SE, Kumar V, Serrano-Mislata A, Madueno F, Krajewski P, Meyerowitz EM (2010). Orchestration of floral initiation by *APETALA1*. Science.

[32] Mao Y, Zhang Z, Feng Z, Wei P, Zhang H, Botella JR, Zhu JK (2016). Development of germ-line-specific CRISPR-Cas9 systems to improve the production of heritable gene modifications in *Arabidopsis*. Plant Biotechnol J.

[33] Yan L, Wei S, Wu Y, Hu R, Li H, Yang W, Xie Q (2015). High-efficiency genome editing in *Arabidopsis* using *YAO* promoter-driven CRISPR/Cas9 system. Mol Plant.

[34] Yanofsky MF, Ma H, Bowman JL, Drews GN, Feldmann KA, Meyerowitz EM (1990). The protein encoded by the *Arabidopsis* homeotic gene agamous resembles transcription factors. Nature.

[35] Kleinstiver BP, Prew MS, Tsai SQ, Topkar VV, Nguyen NT, Zheng Z, Gonzales AP, Li Z, Peterson RT, Yeh JR (2015). Engineered CRISPR-Cas9 nucleases with altered PAM specificities. Nature.

[36] Xie K, Zhang J, Yang Y (2014). Genome-wide prediction of highly specific guide RNA spacers for CRISPR-Cas9-mediated genome editing in model plants and major crops. Mol Plant.

[37] Clough SJ, Bent AF (1998). Floral dip: a simplified method for Agrobacterium-mediated transformation of *Arabidopsis thaliana*. Plant J cell Mol Biol.

