# Supplementary material for: Efficient multiplex mutagenesis by RNA-guided Cas9 and its use in the characterization of regulatory elements in the AGAMOUS gene
Source: Plant Methods. 2016 Apr 25;12:23. doi: 10.1186/s13007-016-0125-7 (PMC4845321; doi:10.1186/s13007-016-0125-7)
Supplement: Supplementary file 1 — 10.1186/s13007-016-0125-7 Expression pattern of the AT4G05320 (UBQ10) gene in Arabidopsis (Fig. S1). a shows spatial and temporal expression pattern of AT4G05320 gene during Arabidopsis seed development and stages of the life cycle and b indicates distribution of expression level of AT4G05320 gene. Heat maps (left) show the normalized microarray expression value according to the color scales shown. Bar chart (right) shows the distribution of expression levels in different tissues and in different development stages. Genotyping of 12 T1 transformants for deleting a 450 bp intron segment in the AGMAMOUS gene (Fig. S2). The size of mutated allele is 450 bp smaller than the wild type allele. Plants #3, #6 and #10 in the black box contain the mutated allele. Primers used in this study and procedure for modifying the CRISPR/Cas9 system and protocol to construct a CRISPR/Cas9 vector. [file 13007_2016_125_MOESM1_ESM.doc]

**Supplemental Figures:**

**
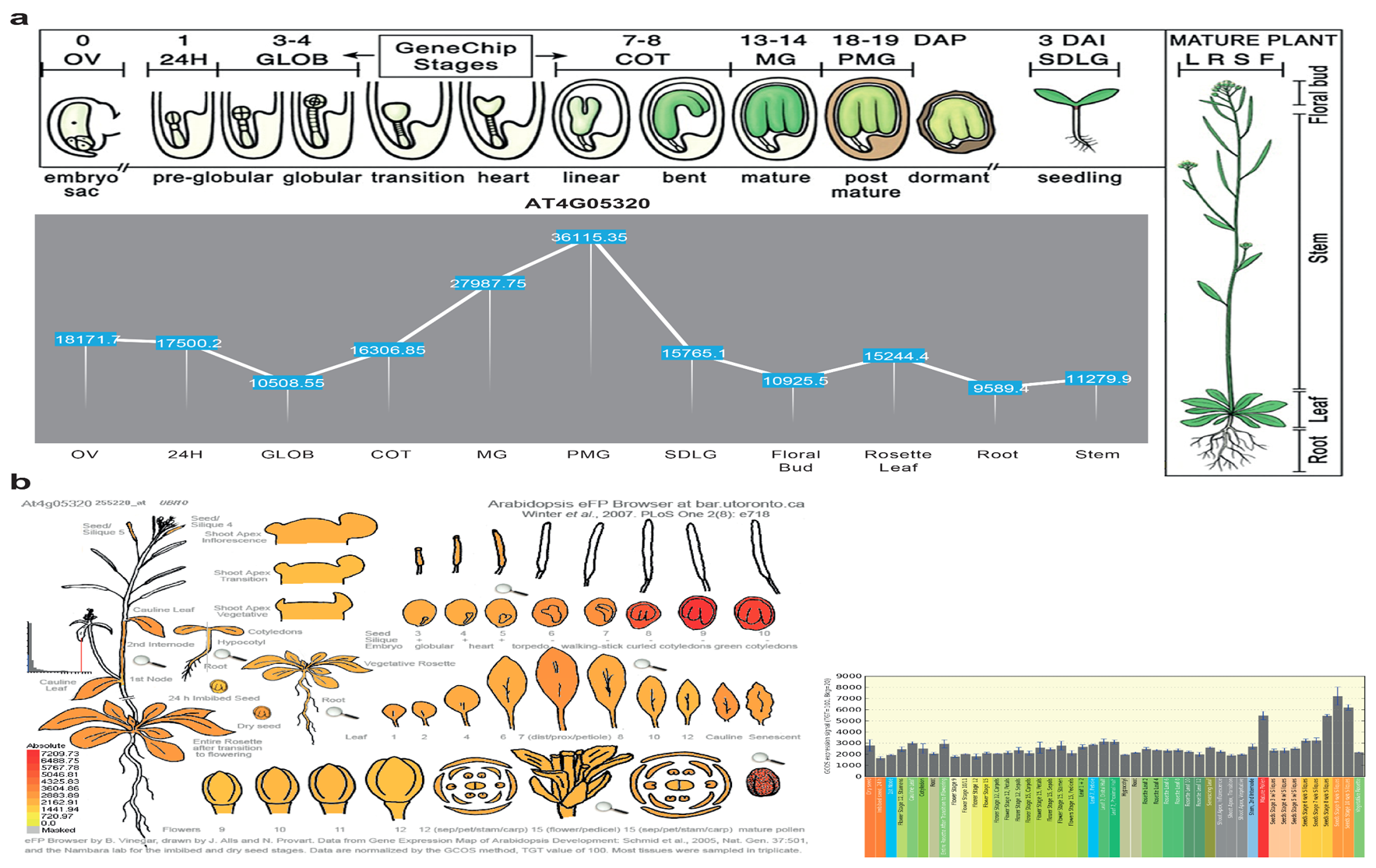
**

**Fig. S1: Expression pattern of the *AT4G05320* gene in *Arabidopsis*.** Spatial and temporal expression pattern of *AT4G05320*gene during Arabidopsis seed development and stages of the life cycle. Gene expression data and schematic representation were obtained from Le et al. . OV, unfertilized ovule; 24H, 24-h postpollination seed; GLOB, globular-stage seed; COT, cotyledon-stage seed; MG, mature-green-stage seed; PMG, postmature-green-stage seed; SDLG, seedling; L, leaf; R, root; S, stem; F, floral buds (a). Distribution of expression level of *AT4G05320gene*. Plots were made with the Arabidopsis eFP Browser (<http://bar.utoronto.ca/efp_arabidopsis/cgi-bin/efpWeb.cgi>) . Heat maps (left) show the normalized microarray expression value according to the color scales shown. Bar chat (right) shows the distribution of expression levels in different tissues and in different development stages (b).


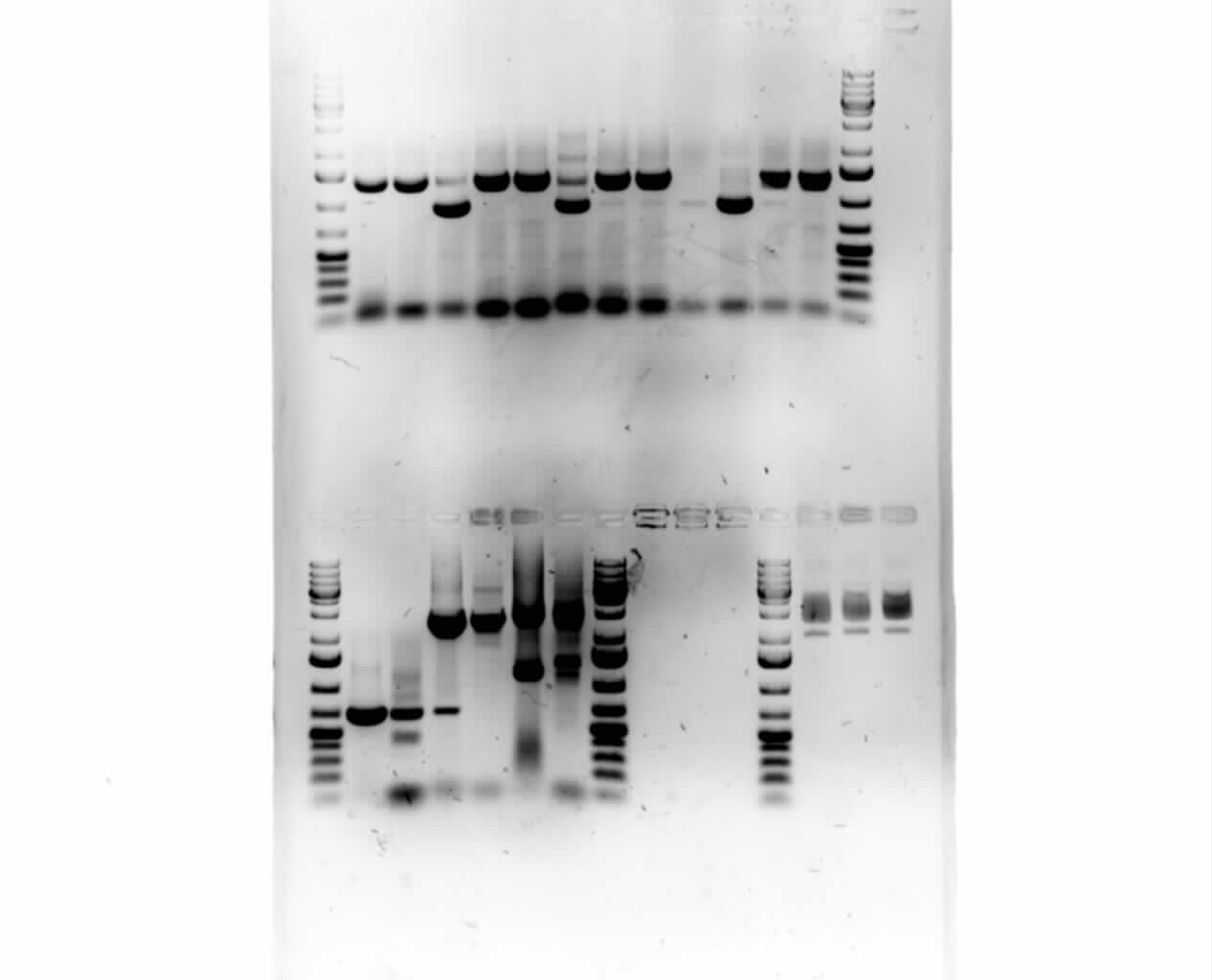


#1

#2

#3

#4

#5

#6

#7

#8

#9

#10

#11

#12

wild type allele

mutated allele

∆ 0.45kb

**Fig. S2: Genotyping of 12 T1 transformants for deleting a 450bp intron segment in the *AGMAMOUS* gene.**The size of mutated allele is 450bp smaller than the wild type allele. Plants#3, #6 and #10 in the black box contain the mutated allele.

**Supplemental Tables**

| **Table S1. Primers used in this study** | | |
| --- | --- | --- |
| **name** | **sequence (5'-3')** | **application** |
| K1178 | TCAGCACTGTCGACCTTGTACCTCTCAATAGTCCC | Genotyping 0.45bkb deletion in *AGAMOUS* 2nd intron |
| K1180 | AGCTAGAGCCTAGGAGAGGTTGGAATTGAGCGCAAG |
| K1531 | TCGTAACTCTAATCGTCGGGTG | Genotyping 5.19kb deletion in *SEP3* |
| K1532 | GTCTAGTTCGATGGACTAGCTTC |
| K731 | GATTGAGATATCGTAGGTATAGCT | Targeting *AP1* introns |
| K732 | AAACAGCTATACCTACGATATCTC |
| K1347 | GATTGTTCTATCTAGGAAATCGAT |
| K1348 | AAACATCGATTTCCTAGATAGAAC |
| K842 | TAGCTCGAGATGGATTACAAGGATGATGATGATAAGGATTACAAGGATGATGATGATAAG | amplifying pcoCas9 plasmid |
| K843 | CACTTTATGCTTCCGGCTCGTATGTTGTGTGG |
| K511 | AACCTGCAGGCTCGACGAGTCAGTAATAAACG | identifying *UBQ10* promoter |
| K512 | TTCTCGAGCTGTTAATCAGAAAAACTCAG |
| K430 | GATTGAGTATGCTAATGACGTGCC | targeting *ELF6* |
| K431 | AAACGGCACGTCATTAGCATACTC |
| K432 | GATTGGAGCCAGTGCGCTATGACG |
| K433 | AAACCGTCATAGCGCACTGGCTCC |
| K434 | GATTGTCTGAGTCTGGCTGCTGCG | targeting *REF6* |
| K435 | AAACCGCAGCAGCCAGACTCAGAC |
| K436 | GATTGCCGATCTGCTGCTGGCGCG |
| K437 | AAACCGCGCCAGCAGCAGATCGGC |
| K440 | GATTGTCATCAACACAGCGCCAGC | targeting *At5g46910* |
| K441 | AAACGCTGGCGCTGTGTTGATGAC |
| K442 | GATTGGGGATTTCCCGATTGCAAC |
| K443 | AAACGTTGCAATCGGGAAATCCCC |
| K838 | GATTGCGCAACTTAGCTGTCGTGT | targeting *SEP3* |
| K839 | AAACACACGACAGCTAAGTTGCGc |
| K1279 | GATTGCAGTCTCACACTATCTATT |
| K1280 | AAACAATAGATAGTGTGAGACTGC |
| K1125 | GATTGCACTCTAATTTTGCCAGCA | tartgeting *AGAMOUS* 2nd intron |
| K1126 | AAACTGCTGGCAAAATTAGAGTGC |
| K633 | GATTGTCTCATTATGGGTACTGAG |
| K634 | AAACCTCAGTACCCATAATGAGAC |
| K1335 | ACTCGGATCTTAAGATTATGAACTCG | Genotyping 0,68 kb deletion in *AP1* |
| K1340 | GGATCATCTTCTTGATACAGACCACTGC |
| K968 | AGCTTTGCCATTGGCACCTGTG | Genotyping 1.88 bkb deletion in *ELF6* |
| K969 | TCAATAGTGTGTTCGAGGCAG |
| K970 | GGTTGCTCCAGAGTTCAGACC | Genotyping 0.27 bkb deletion in *REF6* |
| K971 | CATGGTCCTCCACATGCCAAGC |
| K972 | ACCAACTTCAGCGTCATGTGG | Genotyping 2.0 bkb deletion in *At5g469106* |
| K975 | ATCTTCCTTCGTGATGATGCG |
| K1508 | GCCTTTTCAGAAATGGATAAATAGCCTTGCTTC | Genotyping *ref6-3* mutant |
| K170 | CAGTTGCAACTCTGGAGAAGG |
| K173 | AGGTTTGGATGTCACATCAGG |
| K167 | ACGTCAATGCGGTAATCATTC | Genotyping *elf6-3* mutant |
| K168 | TTTGCAGATCCCATTGCTTAC |
| K150 | ATTTTGCCGATTTCGGAAC |
| K2181 | CGGATCGAGAACACAACGAATCG | Detectimg *AGAMOUS* splicing variant |
| K869 | TGACCCTATCGTCTCACCCATC |
| K2180 | GATGAGTGCGACTTCAGCATCACA | P1 for *AGAMOUS* expression |
| K2181 | CGGATCGAGAACACAACGAATCG |
| K2178 | GTAGATTAAGTTTAGAAAATAGGTAG | P3 for *AGAMOUS* expression |
| K2179 | GCTGATTCTTGTTGATAATACTGTGC |
| K868 | GCACAGTATTATCAACAAGAATCAGCC | P2 for *AGAMOUS* expression |
| K869 | TGACCCTATCGTCTCACCCATC |
| K41 | GTGAAAACTGTTGGAGAGAAGCAA | qPCR primer for AT4G34270 |
| K42 | TCAACTGGATACCCTTTCGCA |
| K1 | TGTAAAACGACGGCCAGT | detecting the presence of T-DNA |
| K197 | CTGTTAATCAGAAAAACTCAG |

**Supplemental Methods**

**Procedure for modifying the CRISPR/Cas9 system and protocol to construct a CRISPR/Cas9 vector.**

Modifying the CRISPR/Cas9 system.

The AtU6-26-SK plasmid for cloning sgRNA expression cassette and 35S-Cas9-SK containing a human codon optimized Cas9 gene in it were kindly provided by Dr. Jiankang Zhu from Shanghai Center for Plant Stress Biology, Chinese Academy of Sciences.AtU6-26 was modified to have unique cutting sites in the order of *Xba*I, *Sbf*I and *Not*I at 3’end of polyT terminator of sgRNA. Meanwhile one *Spe*I site locating at 3’end of polyT terminator was removed. To achieve this, the whole sgRNA expression cassette was amplified by K470 and K677 primer then the PCR product was digested by *Kpn*I and *Not*I and was ligated into the *Kpn*I and *Not*I digested AtU6-26 plasmid. The resulting vector has two adjacent cutting site, *Kpn*I and *Spe*I at 5’ end while two another closely linked cutting sites, *Xba*I and *Sbf*Iat 3’of the sgRNA expression cassette (pAtU6-26::sgRNA-polyT terminator).This plasmid was named AtU-26-V4.

In order to test the efficiency of different Cas9 and promoter combination. The *Xho*I site adjacent to 35S promoter of 35S-Cas9-SK and *Sbf*I site of pCAMBIA1300 were omitted, respectively by simple primer annealing. The pCAMBIA1300 was referred as 1300∆SBF. Coding region of a plant codon optimized Cas9 gene, coupling with Nos terminator was amplified. The PCR fragment was then digested by*Sbf*I and *EcoR*I and was inserted into 35S-Cas9-SK which had been digested by the same enzyme. The newly generated plasmid was termed as p35S-pcocas9-SK.In order to generate pUBQ10-hucoCa9-SK and pUBQ10-pcoCa9-SK, the fragment of UBQ10 promoter was amplified using K511 and K512, containing *Sbf*I and *Xho*I cutting site, respectively. The PCR fragment and both 35S-Cas9-SK and 35S-pcocas9-SK were digested with *Sbf*I and *Xho*I which enabled UBQ10 promoter to replace 35S promoter in both cases. Each of the four Cas9-SK plasmids can be then inserted into 1300∆SBF by digesting the plasmid with *Kpn*I and *EcoR*I.

CRISPR/Cas9 vector construction and infiltration

All the sgRNA primers were designed with the web-tool “CRISPR PLANT” (<http://www.genome.arizona.edu/crispr/>) . All the single sgRNA was made according to the description by *Feng et al*. In short, the AtU6-26V4 plasmid was digested by *Bbs*I and was ligated together with the annealed primer. Primers for sgRNA mentioned in this study was shown in **Supplemental Table 1**.

To combine multiple sgRNA in one single construct, single sgRNA expression cassette needs to be made first. Two independent sgRNA expression cassette can be combined together when one of the sgRNA expression cassette included plasmid is digested by *Kpn*I and *Spe*I while the other plasmid is digested by *Kpn*I and *Xba*I (Fig. 1, panel ②). More sgRNA expression cassettes could be combined together in one plasmid by repeating the same procedure. Then, the combined sgRNA expression cassettes which contain different guide sequences against different loci could be entirely removed by cutting with *Kpn*I and *Sbf*I by which the cas9 expression cassette included binary vector were also digested (Fig. 1, panel ③, ④).The transgenic lines were produced by infiltrating Col-0 using floral-dip method .

**ChIP-seq data**

The binding data of AP1, AP2 and SEP3 to *AGAMOUS* second intron were from

**Supplemental References**

1. Xie K, Zhang J, Yang Y: Genome-wide prediction of highly specific guide RNA spacers for CRISPR-Cas9-mediated genome editing in model plants and major crops. Molecular plant 2014, 7(5):923-926.

2. Clough SJ, Bent AF: Floral dip: a simplified method for Agrobacterium-mediated transformation of Arabidopsis thaliana. The Plant journal : for cell and molecular biology 1998, 16(6):735-743.

3. Kaufmann K, Pajoro A, Angenent GC: Regulation of transcription in plants: mechanisms controlling developmental switches. Nature reviews Genetics 2010, 11(12):830-842.

4. Kaufmann K, Muino JM, Jauregui R, Airoldi CA, Smaczniak C, Krajewski P, Angenent GC: Target genes of the MADS transcription factor SEPALLATA3: integration of developmental and hormonal pathways in the Arabidopsis flower. PLoS biology 2009, 7(4):e1000090.

5. Kaufmann K, Wellmer F, Muino JM, Ferrier T, Wuest SE, Kumar V, Serrano-Mislata A, Madueno F, Krajewski P, Meyerowitz EM et al: Orchestration of floral initiation by APETALA1. Science 2010, 328(5974):85-89.

6. Yant L, Mathieu J, Dinh TT, Ott F, Lanz C, Wollmann H, Chen X, Schmid M: Orchestration of the floral transition and floral development in Arabidopsis by the bifunctional transcription factor APETALA2. The Plant cell 2010, 22(7):2156-2170.
